# Towards Preventing and Managing Conflict of Interest in Nutrition Policy? An Analysis of Submissions to a Consultation on a Draft WHO Tool

**DOI:** 10.34172/ijhpm.2020.52

**Published:** 2020-04-21

**Authors:** Rob Ralston, Sarah E. Hill, Fabio da Silva Gomes, Jeff Collin

**Affiliations:** ^1^Global Health Policy Unit, Social Policy, School of Social and Political Science, University of Edinburgh, Edinburgh, UK.; ^2^SPECTRUM Consortium (Shaping Public Health Policies to Reduce Inequalities and Harm), London, UK.; ^3^Department of Noncommunicable Diseases and Mental Health, Pan-American Health Organization/World Health Organization, Washington, DC, USA.

**Keywords:** Nutrition, Health Governance, Conflict of Interest, Commercial Sector, Global Health

## Abstract

**Background:** With multi-stakeholder approaches central to efforts to address global health challenges, debates around conflict of interest (COI) are increasingly prominent. The World Health Organization (WHO) recently developed a proposed tool to support member states in preventing and managing COI in nutrition policy. We analysed responses to an online consultation to explore how actors from across sectors understand COI and the ways in which they use this concept to frame the terms of commercial sector engagement in health governance.

**Methods:** Submissions from 44 Member States, international organisations, non-governmental organizations (NGOs), academic institutions and commercial sector actors were coded using a thematic framework informed by framing theory. Respondents’ orientation to the tool aligned with two broad frames, ie, a ‘collaboration and partnership’ frame that endorsed multi-stakeholder approaches and a ‘restricted engagement’ frame that highlighted core tensions between public health and food industry actors.

**Results:** Responses to the WHO tool reflected contrasting conceptualisations of COI and implications for health governance. While most Member States, NGOs, and academic institutions strongly supported the tool, commercial sector organisations depicted it as inappropriate, unworkable and incompatible with the Sustainable Development Goals (SGDs). Commercial sector respondents advanced a narrow, individual-level understanding of COI, seen as adequately addressed by existing mechanisms for disclosure, and viewed the WHO tool as unduly restricting scope for private sector engagement in nutrition policy. In contrast, health-focused NGOs and several Member States drew on a more expansive understanding of COI that recognised scope for wider tensions between public health goals and commercial interests and associated governance challenges. These submissions mostly welcomed the tool as an innovative approach to preventing and managing such conflicts, although some NGOs sought broader exclusion of corporate actors from policy engagement.

**Conclusion:** Submissions on the WHO tool illustrate how contrasting positions on COI are central to understanding broader debates in nutrition policy and across global health governance. Effective health governance requires greater understanding of how COI can be conceptualised and managed amid high levels of contestation on policy engagement with commercial sector actors. This requires both ongoing innovation in governance tools and more extensive conceptual and empirical research.

## Introduction

The increasing prominence of multi-stakeholder approaches in global health has highlighted potential tensions between public health goals and the interests of non-state partners. The significance of such conflicts of interest (COI) is a matter of particular salience in non-communicable disease (NCD) policy, given that NCD epidemics are substantially driven by unhealthy commodity industries including alcohol, tobacco and ultra-processed food and drink producers.^1,2^ In the context of emerging evidence about the limited effectiveness of public-private partnerships in global health^3,4^ and public health nutrition^5-7^ the extent to which states can and should engage with commercial and other non-state actors in efforts to address these epidemics has emerged as a central fault line in contemporary health governance.^8^ At a global level, the complex politics across this divide were recently illustrated by the World Health Organization’s (WHO’s) tortuous negotiation of its Framework for Engagement with Non-state Actors (FENSA), the most protracted and fractious aspect of the latest WHO reforms.^9-11^

Debates regarding terms of engagement with the food industry in nutrition governance are particularly complex and contentious.^8,12,13^ In 2012, the WHO’s *Comprehensive implementation plan on maternal, infant and young child nutrition* called on Member States to “establish a dialogue with relevant national and international parties and form alliances and partnerships to expand nutrition actions with the establishment of adequate mechanisms to safeguard against potential conflicts of interest”^14^– thus situating nutrition policy at the centre of such debates. On one hand, the plan epitomises a wide-ranging commitment to public-private partnerships and multi-stakeholder platforms as key mechanisms for the pursuit of health and development goals,^15^ highlighted in the call for revitalized and enhanced partnerships in Sustainable Development Goal (SDG) 17.^16^ On the other, it acknowledges scope for divergence between public health goals and the interests of key economic actors.

Member States subsequently sought support from WHO in reconciling these contrasting imperatives in nutrition policy, with the 2014 World Health Assembly requesting the development of “risk assessment, disclosure and management tools to safeguard against possible conflicts of interest in policy development and implementation of nutrition programmes.”^17^ A process entailing a technical consultation^18^ and informal working groups led to WHO publishing a ‘Draft approach on the prevention and management of conflicts of interest in the policy development and implementation of nutrition programmes at country level’ in 2017.^19-21^ This draft approach (hereafter ‘WHO tool’) centres on a risk assessment tool offering a 6-step methodology intended to support Member States in considering their engagement with non-state actors in the area of nutrition.^20^ While addressing potential risks of conflicts with a broad range of actors, the methodology incorporates a particular focus on commercial interests and assessing alignment with nutrition goals (Box 1).

**Box 1. Key Elements of the WHO Draft Approach (“WHO Tool”) for Prevention and Management of COI in Nutrition Policy**

In September 2017, WHO released an introductory paper,^19^ discussion paper,^20^ and a proposed decision-making process and tool^21^ describing its ‘Draft approach on the prevention and management of conflicts of interest in the policy development and implementation of nutrition programmes at country level.’ (Final versions of these documents were subsequently presented to the WHO Executive Board and the World Health Assembly in 2018,^22-24^ with the discussion paper retitled as a ‘Report by the Director General’). These documents outline the purpose, principles and key elements of the tool, which takes the (non-binding) form of guidance to Member States to be used at their discretion in supporting the development of nutrition policy. The primary focus of the tool is to protect Member State’s goals in relation to nutrition policy, with broader health and other government goals considered as part of the wider context.

**Purpose of Tool**

- The tool is intended to guide Member States “in their engagement with non-State actors and institutions (‘external actors’)… in the development, design, and implementation of public health nutrition policies and programmes.”^22^ In other words, it is designed for internal use by Member States, where nutrition teams (within national Ministries of Health) are assessing whether or not to collaborate with external actors in their nutrition policies and programmes.
- It was developed following a request from Member States for WHO to develop tools that would help them manage potential conflicts of interest in engaging with external actors in pursuit of population nutrition goals.^17^

**Key Concepts and Principles**

- WHO defines COI as a situation “where there is potential for a secondary interest (a vested interest in the outcome of the government’s work in the area of nutrition) to unduly influence … either the independence or objectivity of professional judgement or actions regarding a primary interest (related to the government’s work).”^22^
- The introductory paper distinguishes between *individual* and *institutional* COIs, where individual COI refers to “a private interest (financial, personal, or other non-governmental interest or commitment)” that might interfere with the government’s public health nutrition goals, while institutional COI describes “situations where the interest of non-State institutions… in particular economic, commercial or financial, are not aligned with the government’s public health policies.”^22^
- The documents describe the goal of *policy coherence* as a key principle with relevance to the WHO tool. In this context, policy coherence refers to the extent to which policies across different government sectors (ie, across Health and other Ministries) are aligned or coordinated.^22^
- Other principles underpinning the tool include the need to ensure that engagement between governments and external actors are *appropriate* (ie, will not undermine nutrition goals), *government-led* (ie, the government sets the parameters of the collaboration), and that adequate internal mechanisms are in place to ensure *accountability and transparency* in the Ministry’s engagement with the external actor.^22^

**Six-Step Decision-Making Process**

- The tool sets out a 6-step process via which Member States assess the appropriateness of a proposal collaboration with an external actor, and (if the collaboration goes ahead) establish appropriate parameters and mechanism to ensure any potential COIs are identified and managed.^24^
- The tool is intended for use by a decision-making unit within the Ministry of Health, which gathers relevant information and undertakes due diligence on the external actor; assesses the relevant elements of the proposed collaboration; makes an assessment about the relative risks and benefits of the collaboration; proposes terms of reference for the collaboration; and recommends measures to manage any potential COI.^24^
- Steps 1-3 focus on assessing the appropriateness of a proposed collaboration. Depending on the profile of the external actor, the rationale for the proposed engagement, and the balance of risks and benefits, a recommendation is made regarding whether or not to proceed with the collaboration.
- Where the proposed collaboration is deemed appropriate, steps 4-6 of the tool provide a framework for establishing the parameters of the collaboration and establishing appropriate measures to ensure accountability and transparency. These measures are intended to support ongoing assessment of the collaboration, with the option that the Member State may withdraw at any point if the engagement is no longer deemed appropriate with respect to the government’s nutrition goals.

Abbreviations: COI, conflict of interest; WHO, World Health Organization.

The WHO tool thus constitutes a key innovation in managing COI in NCD governance, arguably comparable in significance to the commitment in Article 5.3 of the WHO Framework Convention on Tobacco Control to protect tobacco control policies from the commercial and other vested interests of the tobacco industry.^25,26^ An important opportunity to examine responses to the tool, and to shed light on the global politics of COI in nutrition policy, was offered by an online consultation WHO held from September 11-29, 2017. Participants were encouraged to provide written feedback on the draft tool and associated introductory and discussion papers, with responses received from across Member States, inter-governmental organizations (IGOs), non-governmental organizations (NGOs) as well as from private sector actors. Based on analysis of these responses, this study aims to explore the differing ways in which COI is understood by diverse actors in global health debates and to consider the relevance of these varying conceptions to their appraisals of the WHO tool and their preferences regarding the role of commercial actors in health governance. In doing so, it draws on the distinction between *individual*and *institutional* COI used in the WHO tool (Box 1) and reflected in the academic literature.^15,16^

## Methods

WHO’s online consultation was open from September 11-29, 2017, with respondents encouraged to provide written feedback on the draft tool and the associated introductory and discussion papers. Submissions were made by Member States,^6^ UN agencies and other IGOs,^5^ NGOs,^12^ academic institutions,^7^ and commercial sector actors^14^ (see Table 1). All submissions were available in English except the Member State submission from Colombia and a submission from the Mexican Council of Consumer Product Industries (CONMéxico), which were both in Spanish and were reviewed by one of the authors (FSG) for the purposes of analysis.

**Table 1:** Respondents to the WHO Consultation by Overall Position (Supportive, Critical or Unclear) and Actor Type

**Supportive of WHO Tool**
**Member States**
Brazil
Canada
Colombia
Namibia
**UN agencies and other IGOs**
UN Network for SUN Secretariat*
WFP
**NGOs**
Consumer Council of Fiji
FHI360/Alive and Thrive Southeast Asia
Healthy Food Alliance, Brazil
Healthy Latin America Coalition
The Cochrane Collaboration, United Kingdom
UK Health Forum, United Kingdom
European Public Health Alliance
Third World Network
**Academic institutions**
Academia Española de Nutrición Humana y Dietética, Spain
NNEdPro Global Centre for Nutrition and Health, United Kingdom
Niger Delta University, Nigeria
Deakin University, Australia
University of Cambridge, United Kingdom
University of Sydney, Australia
National Institute for Health and Welfare, Finland
**Critical of WHO Tool**
**Member States**
The United States
**UN agencies and other IGOs**
SUN Secretariat*
**NGOs**
Geneva Infant Feeding Association, Switzerland
International Baby Food Action Network
World Public Health Nutrition Association
**Commercial sector entities**
AFH, the United States
CONMéxico, Mexico
EAGL, the United States
FIA
Global Dairy Platform, United States of America
GMA, the United States
Council for International Business, the United States
International Council of Beverages Associations
International Special Dietary Foods Industries
Private Sector Mechanism of the CFS
IDF
International Council of Beverages Associations
Federalimentare - Italian Food & Drink Industry Federation, Italy
Nutrispectives, LLC, the United States
**Position Unclear/Mixed**
**Member States**
New Zealand
**UN agencies and other IGOs**
OECD
UNDP
**NGOs**
Centre for Health Science and Law, Canada
**Academic institutions**
NNEdPro Global Centre for Nutrition and Health, United Kingdom

Abbreviations: WHO, World Health Organization; SUN, Scaling Up Nutrition; WFP, World Food Programme; NGO, non-governmental organization; AFH, Alliance for Food & Health; CONMéxico, Consejo Mexicano de la Industria de Productos de Consumo; EAGL, Engaging America’s Global Leadership; FIA, Food Industry Asia; GMA, Grocery Manufacturers Association; CFS, Committee on Food Security; IDF, International Dairy Federation; UNDP, United Nations Development Programme, OECD, Organisation for Economic Co-operation and Development; IGOs, inter-governmental organizations ^a^ Note that contrasting submissions were made by two different actors affiliated to the SUN partnership, namely the UN Network for SUN Secretariat and the SUN Secretariat.

This paper takes a constructivist approach, identifying the various ‘policy frames’ developed by actors to shape debates about COI. This analytical perspective focuses on how actors construct or interpret issues in ways that influence how policy problems are defined, who is considered a legitimate stakeholder, and possible solutions. The politically contested nature of health governance means that different groups of actors compete for their interpretation to become the dominant framing. Findings from public health research on framing^27-30^ suggest that commercial sector actors strategically use frames as a ‘weapon of advocacy’^31^ to promote policies that are aligned with their economic and political interests. We undertook analysis of all 44 submissions, focusing particularly on respondents’ framing the WHO tool, and ideas about COI. Each submission was reviewed by 2 authors and findings from the frame analysis were used to assign the respondent’s overall position in relation to the tool, ie, broadly supportive, broadly critical, or unclear/mixed.

Based on this approach, we identified 2 policy frames around which actors’ interpretations of the tool are structured in relation to their (*i*) overall orientation to the tool, (*ii*) conceptualization of COI, and (*iii*) concerns with specific elements of the tool. Table 2 provides a generalised representation of problem definitions and policy solutions used within these 2 frames. These labels delineate 2 competing representations evident in submissions: ie, *collaboration and partnership* frames that prioritize partnership and multi-sectoral approaches and are sceptical about the relevance and appropriateness of the tool in global health governance; and *conflict and restricted engagement* frames which see nutrition policy as defined by core tensions between public health and (at least some) food industry interests and favour development of a powerful tool to address perceived inadequacies of existing governance arrangements in managing COI.

**Table 2:** Policy Frames, Definitions and Solutions in Submissions to the WHO Consultation

**Policy Frame**	**Problem Definition**	**Policy Solution**
Collaboration and partnership	Draft WHO tool identified as incompatible with commitments to partnership and multi-stakeholder approaches in SDG agenda	COI seen as minimal, applying equally across all non-state actors and as being adequately managed by existing practices
Conflict and restricted engagement	Nutrition policy characterised by tensions between public health goals and economic interests	COI concerns focus on more effectively managing and delineating terms of engagement with commercial sector actors. WHO tool should address institutional and/or structural conflicts of interest

Abbreviations: WHO, World Health Organization; SDG, Sustainable Development Goal; COI, conflict of interest.

## Results

While all 44 respondents stated their general agreement in principle with a need to address COI, submissions varied significantly in their support for a WHO-produced tool to address COI in nutrition policy (Table 1). The tool was widely supported by Member States, many NGOs, and academic institutions. Commercial sector entities were all highly critical, as was the Member State submission from the United States and that from the Secretariat of the Scaling Up Nutrition (SUN) partnership. From a very different perspective, a small number of NGOs saw the WHO tool as incapable of addressing the challenge of preventing COI, while 5 submissions did not exhibit a clear overall position on it (Figure).

**Figure:**
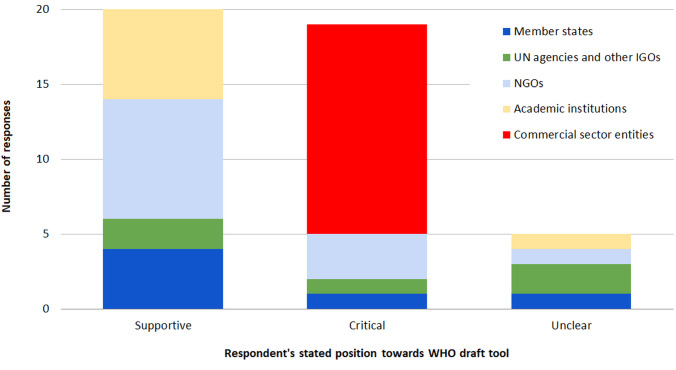
Numbers of Supportive, Critical and Unclear Responses to the WHO Consultation, by Actor Type. Abbreviation: WHO, World Health Organization.

### Policy Frames

#### Collaboration and Partnership

A ‘collaboration and partnership’ frame was consistently articulated across submissions made by commercial sector actors, by the Member State submission from the United States, and by the Secretariat of the SUN partnership, all of whom used the consultation to undertake an extensive critique of the tool. This strategic framing was strongly critical of the draft tool as exclusionary and distrustful in its attention to the commercial sector; consistently rejected any comparison between food and tobacco industries or scope for lesson learning from practice in tobacco control; viewed the tool’s recommendations as inconsistent with principles of good governance, and particularly with the SDG agenda; and presented COI as being both compatible with extensive engagement and as being satisfactorily addressed by existing practices.

Using identical phrasing, both the Italian Food and Drink Federation and the Private Sector Mechanism to the UN Committee on Food Security (CFS) critiqued the language of the document as revealing “categorical and unhelpful distrust of any private sector actor” and as “denigrating industry.”^32^ In similar vein, Food Industry Asia called on WHO to reconsider its proposals, “especially those that are designed to shut out the private sector from any meaningful policy discussion,”^33^ while the submission from the US government noted:

*“We are deeply concerned by the overarching tone of exclusion present in the draft documents, which runs directly counter to the current global approach of inclusiveness and recognition of the need for all stakeholders to work together to achieve global nutrition goals.*”^34^

This concern also entailed a rejection of lesson drawing from experiences in managing COI within tobacco control. For the US government, “blanket consideration of the food and beverage sector in the same manner as the tobacco industry is inappropriate,”^34^ the Private Sector Mechanism to the CFS rejects “inappropriate comparisons between the food and beverage industries and the tobacco industry,”^32^ comparisons which the Italian Food and Drink Federation further regards as “unacceptable.”^35^

More broadly, submissions using the ‘collaboration and partnership’ frame presented the tool’s potential constraints on private sector engagement as incompatible with established norms of good governance, including within WHO and particularly across the SDG agenda. This centred on the claim that the draft tool diverged markedly from the stated intention of ensuring consistency with “WHO’s overall policies and practices,”^20^ with submissions from Food Industry Asia and the International Food and Beverage Alliance strategically positioning the tool as counter to the “spirit” of FENSA negotiations.^33,36^ The Grocery Manufacturers Association (GMA) contended that “no tool recommended by the WHO should have exclusionary criteria […] which are not consistent with FENSA.”^37^ This framing is also evident in the United States submission, which notes:

*“Problematic language […] in the proposed decision-making process and tool advising where “great caution” should be exercised. FENSA states WHO will exercise “particular caution” when engaging with private sector entities.*”^34^

Private sector actors frequently invoked a claimed disjuncture between the tool’s cautious approach to engagement with the private sector and the UN commitment to multi-sectoral and partnership approaches as preferred mechanisms for advancing the SDGs. Indeed, commercial sector actors accounted for over 85% of the total number of references to the SDGs across all submissions. Their problem definition focused on perceived challenges to the effectiveness and accountability of health governance, portraying the tool as frustrating the potential of partnerships with the private sector in achieving goals across nutrition, global health and sustainable development:

International Council of Beverages Associations: *“The theme contained in these documents – namely, that the input of the private sector on topics related to nutrition policies is not only unwelcome but should be subject to near-automatic exclusion […] stands in contrast to high-level global engagement approach [sic] endorsed elsewhere […] This exclusion runs counter to the approach endorsed by the UN General Assembly in its pursuit of the SDGs.*”^38^

Alliance for Food and Health: “*These documents raise concerns that Member States may significantly restrict engagement with one of the non-State [sic] actors: the private sector. […] Restricted engagement appears contradictory to SDG 17; specifically it can weaken attempts to provide sustainable solutions to the NCD crisis by excluding a key stakeholder group.*”^39^

This assertion of the value of extensive private sector engagement in nutrition policy is coupled with a rejection of any claim that intrinsic tensions or distinctive issues are entailed by their participation. The Private Sector Mechanism to the CFS noted that “(c)ommercial motives are not incompatible with public health interests,”^32^ a point phrased as “not inherently incompatible” by the Italian Food and Drink Federation,^35^ while several submissions shifted focus towards conflicts of interest they identified among other non-state actors. Food Industry Asia and the US-based GMA identically argued that relevant questions for assessing conflicts “relate not to an actor’s sector but rather to the transparent management of inputs and outcomes in policy considerations.”^33,37^ In extending attention to conflicts beyond the private sector, the International Dairy Federation note that “(s)ources of bias are extensive and complex” and cite “many types of bias including cognitive, financial, publication, statistical, political, ethical, philosophical, etc”^40^; the Global Dairy Platform similarly contend that “statements in publications, history of unpaid advisory roles, and organisational affiliations” may be as significant as financial interests.^41^

The International Dairy Federation is the only private sector actor to explicitly include a definition of COI in its submission, citing a 2009 Institute of Medicine report^42^ to which it attributes the designation, echoing Thompson,^43^ of “a risk that professional judgment or actions regarding a primary interest will be unduly influenced by a secondary interest.”^40^ More broadly, the literature cited in these submissions contesting the tool’s handling of COI reaffirms the emphasis on individual level concerns alongside extending the range of relevant conflicts beyond the private sector. The Global Dairy Platform cites a paper that uses the term “white hat bias” to describe a tendency among health-oriented researchers to distort “research-based information in the service of what may be perceived as righteous ends.”^41^ The Alliance for Food and Health^39^ cites a paper by Rowe et al for the Conflict of Interest Working Group of the International Life Sciences Institute North America (a food industry founded and led organization) on criteria for selecting participants for expert advisory committees.^44^ Principles developed by the International Life Sciences Institute working group also formed the basis of a report on addressing COI in nutrition research^45^ cited by both the Global Dairy Platform and the International Dairy Federation.^40,41^

The preferred policy solutions associated with such perspectives centre on positioning COI as a legitimate but marginal concern that can be effectively addressed by established practices regarding disclosure. The International Dairy Federation contends that “simply being aware of the interests of the [non-state actor] can ensure any inappropriate input … would be more easily recognizable,”^40^ while the GMA suggest that “transparent disclosure standards and clear, equitable processes can identify, manage and resolve potential conflicts of interest appropriately.”^37^

This perception of challenges posed by COI as being peripheral and manageable is linked to the assertion that the identification of COI should not narrow the scope of engagement by commercial actors in nutrition policy. The Italian Food and Drink Federation and the Private Sector Mechanism to the CFS both argue that it “is unfair to allow a perception of COI to affect decisions on whether to continue to engage with a particular private sector actor,”^32,35^ while the Alliance for Food and Health suggest that governance mechanisms to identify interests and objectives “should serve as a mechanism to enable, rather than prohibit, partnerships with non-State actors,”^39^ with identical phrasing being used by the SUN secretariat.^46^ The emphasis on the appropriate aim of COI tools as being to promote and enable participation rather than restricting engagement is similarly evident in the submission from the US government:

*“Issues around actual COIs should begin with an intention to bring in stakeholders to work towards common public health goals. COI mitigation and management tools, such as Codes of Conduct, can be used to increase transparency and reduce risk around actual COIs while promote successful multi-stakeholder partnerships.*”^34^

Overall, it is clear that proponents of the ‘collaboration and partnership’ frame consistently reject the idea that tensions exist between the interests of commercial sector actors and public health, and, therefore, that new mechanisms would be required to address COI. Reflecting this, problem definitions emphasize a claimed dysfunctionality of the proposed tool within the wider development agenda, which are then combined with policy solutions that divert attention from corporate actors.

### Conflict and Restricted Engagement

In contrast, proponents of a ‘conflict and restricted engagement’ frame, consisting of several Member States, the majority of participating NGOs, and most academic institutions, perceived the draft WHO tool as offering a potentially important step in strengthening nutrition governance by addressing COI. This positive appraisal is apparent in several actors’ assessments of the decision-making tool, where problem definitions associated with this frame include a particular focus on tensions between commercial interests and public health goals. Recognition of such tensions is central to support for the tool as articulated in the submissions from Brazil, Colombia and Namibia:

*Brazil: [T]he document under online consultation should consider … potential conflicts of interest and forms of control regarding different non-governmental actors participation, particularly food industries and the organizations financed by them. The document should be sensitive to immediate strategies or interventions, based on infant formulas, fortified foods and micronutrient supplements distribution (often disassociated from the local food culture and habits and provided by the companies that manufacture these products, ie direct COIs with the interventions and possible alternatives)*.^47^

The submission by Colombia also emphasized such conflicts as impacting on public health, citing marketing of breast-milk substitutes, sports sponsorship by soda companies, and the revolving door via which government officials later take up positions in the food industry.^48^ Namibia’s Minister of Health and Social similarly described nutrition marketing as one of the “concealed battle fields as far as commercial interest[s] are concerned,” with a tendency for such interests to “always end up being the winner.” This highlighted the importance of WHO guidance “to ensure that the public and mostly the vulnerable are protected at all times against COI” including by actors that collaborate with WHO.^49^

Several submissions deploying a ‘conflict’ frame - including those from Canada, OECD, UN Development Programme and the Centre for Health Science and Law^50-52^ – understood COI in more institutional terms – that is, as a potential conflict between the primary interest of an organisation or collaboration (such as promoting healthy nutrition) and its secondary interests (such as securing financial support). Some actors also questioned the clarity of the documents in defining and presenting COI; comments from a professor at the University of Sydney suggest that the documents “confuse COI with bias,”^53^ while the International Baby Foods Action Network (IBFAN) suggest that draft documents “produce an incorrect understanding of what COI regulation is.”^54^ Yet, although it is clear that COI was conceptualized in varying ways, these organisations shared a broad recognition of such conflict as constituting a significant problem for global heath governance, and one that centred on a perceived need to address tensions between commercial sector interests and public health goals. While acknowledging that COI could arise for any actors engaged in the policy process, the Healthy Latin America Coalition epitomised a widely held (if varyingly expressed) emphasis on issues associated with private sector engagement in nutrition policy:

*“While COI might emanate from many different sectors, including government agencies, the significant role of the ultraprocessed food and beverage industry, and its front groups, is such that it might require special treatment in this proposed process.*”^55^

Food researchers from the University of Cambridge welcomed the tool’s assertion of the primary authority of national governments to develop nutrition policy “and the clarification that while private sector stakeholders can be consulted in meetings, they should be excluded from actual decision-making because of the potential COI.”^56^

Several submissions from NGOs sought to move beyond institutional understandings of COI. This is most explicit in Healthy Food Alliance Brazil calling for the tool to “develop a broader recognition of systemic or structural COI.”^44^ This noted that institutional approaches to managing COI “are often specified very narrowly with respect to the activities of a specific engagement or partnership, and can ignore wider tensions across other spheres of public health,” citing the example of the Global Fund’s justification of a partnership with a leading brewery.^44^

More broadly, the perception of nutrition policy as being characterised by intrinsic or fundamental conflicts between key private sector actors and health goals is strongly evident in several submissions from health-oriented NGOs, exemplified by the European Public Health Alliance:

*[T]he paper and tool addresses issues that the European Public Health Alliance and public health researchers and organisations have long identified; that there is an inherent COI for producers of health-harmful products, including food and drink products, which incentivises them to intervene in policy making with the aim to derail and delay public interest policies, programmes and measures.* ^57^

While all respondents employing the ‘conflict’ frame were supportive of the intent of the draft tool, some groups did raise concerns about its ability to reconcile effectively addressing COI with commitments to partnerships and multi-stakeholder approaches. In arguing for an extension to producers of unhealthy food and drinks of the type of exclusionary approach that WHO adopts with the tobacco industry, the World Public Health Nutrition Association contend:

The text reflects a contradiction between an effort to safeguard policy and programming endeavours in nutrition and a simultaneous reaffirmation of flawed multi-stakeholder and public-private partnerships initiatives… [that] have COI intrinsically built- into them that go against decision-making in the public interest.^58^

In similar vein, IBFAN suggest that the draft documents fail to address the risks of partnership and multi-stakeholder approaches that “blur the lines between public and private and create difficulties for national governments when attempting to protect citizens from undue influence.”^54^ The Geneva Infant Food Association extends this critique of WHO’s proposed approach as “[r]edefining of the COI concept to serve the [multi-stakeholder initiative/partnership] paradigm.”^59^

## Discussion

Analysis of submissions on the WHO tool for managing COI in nutrition policy suggests respondents drew on a range of conceptualisations of COI, ranging from more individualised framings (reflected in many commercial sector submissions) to more structural understandings (most evident in submissions from several health-oriented NGOs). While variation is evident within broad groups of respondents, some key themes emerge from this analysis. These serve to highlight the centrality of competing conceptions of COI to debates regarding nutrition policy and global health governance, and in particular to defining the terms and parameters of appropriate engagement with non-state actors amid concerns about the effectiveness of partnership approaches.^3-7^

The extent to which respondents supported the WHO tool was closely linked with how they conceptualized COI. The diverse range of actors who were generally supportive of the tool typically advanced a broad understanding of COI, albeit with some implicit or explicit variation across institutional and structural conceptions. In contrast, participating organisations who largely rejected the draft tool as inappropriate or unhelpful, notably comprising all of the submissions from commercial actors, often invoked a more individualised understanding of COI. The latter typically presented conflicts as being of limited significance, as adequately addressed by existing widespread practices such as disclosure, and therefore questioned the legitimacy or value of the proposed tool.

Crucially, the ways in which respondents framed COI in nutrition policy is closely linked to their organisation’s preferences in relation to the role of commercial actors in health governance. The analysis of these submissions, therefore, captures key tensions within contemporary health governance, ones that are experienced particularly acutely in nutrition policy. Importantly, the analysis highlights the policy salience of debates around COI, and indicates how advancing such debates might inform efforts to address wider fault-lines in global health and sustainable development. Analysis of the submissions illustrates the challenges confronting the WHO tool given the polarisation of perspectives across participating organisations, and the high levels of contestation surrounding the very concept of COI. Yet the very centrality of COI to broader debates in health governance also highlights powerful opportunities associated with this innovative new instrument as a route towards promoting policy coherence in nutrition and NCD policies (ie, in pursuing increased alignment and coordination of policies across different areas of health governance, and between health and other state sectors). Leveraging such opportunities will require that approaches to applying the tool be adaptive, enabling its further development to build support, encourage uptake, and ensure that it is responsive to diverse national contexts.

Discussing the submissions with reference to contrasting ‘*Collaboration and partnership’* and ’*Conflict and restricted engagement’* frames highlights the substantive divergence across both problem definition and policy solutions. Commercial sector submissions to the online consultation, along with those of the United States and the SUN Secretariat, saw relevant COIs as being distributed across all actors, rejecting the idea that there was anything particularly different or significant about such conflicts in relation to commercial actors, and, in particular, any suggestion that such conflicts should circumscribe the terms of their participation in nutrition policy. This position buttressed the presentation of commercial organisations as legitimate and necessary actors within global health governance, with their claim to central roles in efforts to advance to nutrition, global health and sustainable development presented as endorsed by the UN in the SDGs and by WHO via FENSA.

In contrast, many NGOs and most Member States presented efforts to manage COI with commercial actors as essential to the development of effective nutrition policy. These submissions typically presented the interests of many commercial producers as intrinsically conflicting with public health goals, thus necessitating the development of clear and detailed guidelines for limiting engagement with (or excluding such actors from participation in) nutrition policy. This polarised divide with respect to COI in nutrition policy closely mirrors debates about the terms of engagement of non-state actors in health policy more broadly, notably in the protracted and contentious negotiations within WHO in developing FENSA.^9-11^

In delineating the different ways in which COI is presented and understood across these submissions, the account presented here demonstrates the high levels of contestation to which this concept is subject. While the wider literature has recognised the ambiguity and malleability of COI,^60^ this analysis of the online consultation demonstrates that the variation across individual, institutional and structural understandings has substantive policy significance. Variation in problem definition, or in how COI is understood, corresponds in large part with preferred policy solutions regarding whether and how key commercial sector actors should be engaged in nutrition policy at Member State level.

A further significant, if largely latent, element of contestation is exposed via perspectives on the tool’s aims in both *preventing* and *managing* COI in nutrition programmes at country level. Here there are important differences in positions articulated across civil society submissions, partially cutting across the polarity of the divide between ‘collaboration’ and ‘conflict’ frames. Those NGOs who are most sceptical about the likely effectiveness of the WHO tool, namely IBFAN, Geneva Infant Food Association, and World Public Health Nutrition Association, do so on the basis of a fundamental rejection of the legitimacy or efficacy of multi-stakeholder and partnership governance models; prevention of COI is viewed here as requiring the complete exclusion of commercial sector actors from policy-making, along lines comparable to tobacco control, with the aim of ‘managing’ COI in relation to industry engagement presented as naïve or contradictory.

The task of both preventing and managing COI in nutrition policy encapsulates the dynamics of the wider policy context in which the tool was developed. By contrast with the exclusionary politics of the Framework Convention on Tobacco Control, predicated on an unequivocal identification of incompatibility between health goals and tobacco industry interests,^19^ the WHO tool is explicitly charged with the task of pro-actively identifying and addressing tensions in multi-stakeholder and partnership approaches to nutrition. It represents an attempt to advance the development of “alliances and partnerships” via the “the establishment of adequate mechanisms to safeguard against potential conflicts of interest.”^3^ Thus, some civil society critics dismiss the tool as legitimating public-private partnerships; while – conversely - commercial sector submissions view the tool as hostile to their engagement and as incompatible with the SDG agenda and Goal 17. The majority of submissions, however, clearly welcomed the draft tool as an attempt to begin to ‘square the circle’ inherent in commitments to multi-sectoral approaches in nutrition policy, recognising tensions between health goals and the economic interests of the global food and drinks industry. Rather than being incompatible with SDG 17, the draft tool can therefore be seen as an important innovation in seeking to enhance policy coherence for sustainable development,^61^ recognising that the SDG agenda provides scope for regulation of the commercial determinants of health.^62^

The highly contested politics of COI and the terms of engagement with commercial actors in nutrition policy inevitably shape the contours of the WHO tool and the context of its further development and potential implementation by Member States. When the tool was presented to the Executive Board in January 2018, it was presented as “a living document to be revised according to Member States’ needs and the evolution of engagement with external actors.”^63^ A subsequent informal technical consultation with Member States highlighted ongoing substantive variation in their perceived needs and preferences for participation with the private sector and food industry.^64^ While some states had chosen to restrict such interactions in the development of national nutrition guidelines, this had been operationalised in varying ways, while others expressed concern about whether implementation of the tool would jeopardise any engagement with the private sector.

Member States clearly recognise the need for further evolution of the WHO tool to enhance their ability to navigate such complexity, with suggested amendments including the development of a simplified version of the tool as an initial scoping device; clarification of criteria for exclusion and participation; more specific guidance for managing COI in the context of partnerships; and wider technical assistance to enhance governmental capacity in this sphere.^64^

A key limitation of our study is that data are limited to those actors that responded to WHO’s online consultation, which took place over a very short time-frame (September 1-29, 2017); thus we do not have access to the views of those countries or organizations that did not submit written responses. Of 55 responses, 14 came from commercial actors, 12 from NGOs, 8 from academic institutions, and just 6 from Member States (with a further 5 from UN or other international agencies) – suggesting some sets of actors are better represented in these submissions than others. This should be borne in mind when interpreting count-based data (eg, Figure), which should not be interpreted as representation of the views of all relevant actors (indeed, our data are likely skewed by the large proportion of submissions coming from commercial actors). The extent to which relevant actors respond to such consultations depends in part on capacity and available resources, which are particularly limited for low- and lower-middle income countries. This may explain why – of 6 Member State responses – 3 were from high-income and 3 from upper-middle income countries, with no responses from lower or lower-middle income countries.^65^ We also note that the consultation documents were available only in English, which is likely to have presented an additional barrier to participation from Member States and other actors in regions where English is less commonly used. A more comprehensive assessment of the response of (in particular) Member States to the WHO tool would ideally draw on other data sources (including participation in Member State consultations and technical meetings hosted by the WHO).^18,64^

## Conclusion

The WHO tool for preventing and managing COI in nutrition policy represents an important innovation in global health governance, offering a framework for assessing and managing potential conflicts between public health goals and the interests of the commercial sector and other external actors. This is an important development in global health, demonstrating how consideration of COI can be incorporated in NCD governance beyond tobacco control, and offering Member States support and guidance in safeguarding their nutrition goals in the context of engagement with non-State actors.

Realising the potential of the WHO tool will require a strategic approach to learning lessons from efforts to test its applicability across diverse country contexts, building on the initial experience of Brazil.^66^ There is also a clear need to strengthen research to more effectively support such a process, highlighted by the contestation and confusion of concepts that characterize the submissions analysed here. Analyses of efforts to implement the WHO tool could also address the dearth of empirical studies of initiatives to prevent and/or manage conflicts of interest in health policy contexts.^67^

Submissions on the WHO tool also illustrate how contrasting positions on COI are central to understanding broader debates around the role of commercial sector actors in nutrition policy and across global health. These debates are often dominated by somewhat crude binary categories of partnership with or exclusion of commercial entities; governance innovations such as the tool offer the potential to move past a blanket acceptance or rejection of partnership to identify specific actors and forms of engagement where conflicts of interest can be managed in ways that protect public health nutrition goals.^68^ In this context, the tool may offer a useful reference point for Member States, officials and policy-makers who are concerned to prevent pursuing collaborations which they view as inconsistent with their nutrition goals. This may be particularly helpful amid broad pressures from within and beyond governments to pursue partnerships or to accept offered funds or support. Central to the task of defining appropriate terms of engagement with the private sector in nutrition policy is the need to better differentiate between actors within the ‘food industry,’ an unhelpfully sweeping category that groups together such diverse entities as community-based farming cooperatives and multi-national companies, thus obstructing attempts to differentiate between those actors whose economic interests can and cannot be substantively reconciled or aligned with public health goals.^23^ There is also a pressing need for the development of a more detailed typology of COI that can be operationalised and applied in diverse policy contexts.

## Acknowledgements

Support for this work was provided via a University of Edinburgh ESRC Impact Acceleration Account, grant no ES/M500380/1. The research also draws on related consultancy work on nutrition governance undertaken for WHO (JC, SH, RR) and the Pan American Health Organization (PAHO), Washington, DC, USA (JC, SH). RR, SH and JC are also part of the SPECTRUM Consortium (Shaping Public Health Policies to Reduce Inequalities and Harm), London, UK (MR/S037519/1) supported by the UK Prevention Research Partnership (UKPRP).

## Ethical issues

Ethical review of this research was carried out in accordance with the ethical review processes of the School of Social & Political Science, University of Edinburgh, under which the research was assessed as level 1 (low risk).

## Competing interests

Authors declare that they have no competing interests. The authors are solely responsible for the opinions, hypotheses and conclusions or recommendations expressed in this publication, and they do not necessarily reﬂect WHO nor PAHO’s vision.

## Authors’ contributions

JC conceptualised the study; RR developed the coding framework and undertook data analysis; RR, SEH and JC wrote the first draft; all authors contributed to the writing of the manuscript and agree with its results and conclusions. FSG is a staff member of the PAHO. The authors alone are responsible for the views expressed in this publication, and they do not necessarily represent the decisions or policies of PAHO.

## Authors’ affiliations

^1^Global Health Policy Unit, Social Policy, School of Social and Political Science, University of Edinburgh, Edinburgh, UK. ^2^SPECTRUM Consortium (Shaping Public Health Policies to Reduce Inequalities and Harm), London, UK. ^3^Department of Noncommunicable Diseases and Mental Health, Pan-American Health Organization/World Health Organization, Washington, DC, USA.

## Key Messages

**Implications for policy makers**

- Analysis of submissions across sectors highlighted significant divergence in how conflict of interest (COI) is understood, with important implications for nutrition governance and the roles of non-state actors.
- Commercial sector actors saw COI as effectively addressed by requirements for individual disclosure, and opposed the tool as inhibiting partnership approaches.
- Civil society organisations and most participating member states highlighted broader potential conflicts between food industry interests and public health goals, and largely viewed the tool as an important innovation in seeking to manage and prevent such tensions.
- The centrality of COI to key debates in global health governance highlights the potential contribution of the tool in promoting policy coherence across nutrition and non-communicable diseases (NCDs).

**Implications for public**

The question of how to manage conflicts between commercial sector interests and public health goals is among the most contested in global health. The World Health Organization (WHO) has developed a tool to prevent and manage conflict of interest (COI) in nutrition policy, and responses across governments, commercial sector actors and civil society highlight major divisions in how actors understand such conflicts and their implications for health policy. The tool represents an important innovation in developing effective responses to the problems of obesity and under-nutrition.
